# Supplementary material for: Comprehensive Annotation and Functional Exploration of MicroRNAs in Lettuce
Source: Front Plant Sci. 2021 Dec 24;12:781836. doi: 10.3389/fpls.2021.781836 (PMC8739914; doi:10.3389/fpls.2021.781836)
Supplement: Supplementary file 3 [file Data_Sheet_9.ZIP › GSM2230751.plot/Lsa-miR396e_Lsat_1_v5_gn_8_125940.1_462_TPlot.pdf]

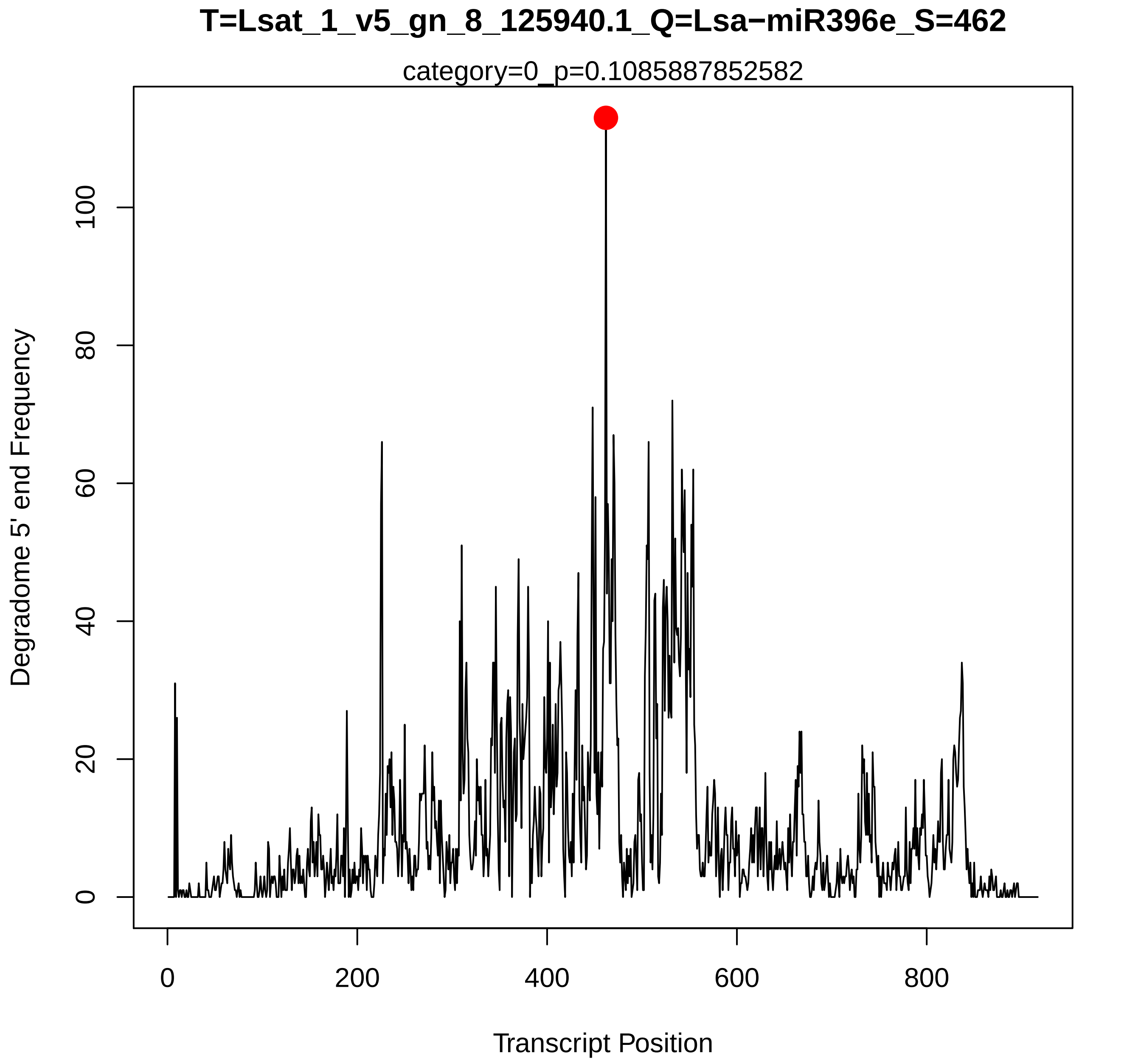
T=Lsat_1_v5_gn_8_125940.1_Q=Lsa−miR396e_S=462
category=0_p=0.1085887852582
Degradome 5' end Frequency
0
20
40
60
80
100
0
200
400
600
800
Transcript Position
